# Pre-departure PCR testing of travellers for SARS-CoV-2 was an effective tool in limiting transmission in Greenland during the early phases of the COVID-19 pandemic

**DOI:** 10.1186/s12889-025-21844-y

**Published:** 2025-02-12

**Authors:** Mie Møller, Kasper Sommerlund Moestrup, Trine Abelsen, Peter Vedsted, Kåre Mølbak, Anders Koch

**Affiliations:** 1 Institute of Health and Nature, University of Greenland, Manutooq 1, Nuussuaq, Nuuk, 3905 Greenland; 2 Department of Veterinary and Animal Sciences, University of Copenhagen, Copenhagen, Denmark; 3 Department of Infectious Disease Epidemiology and Prevention, Statens Serum Institut, Copenhagen, Denmark; 4 Department of Infectious Diseases, Rigshospitalet University Hospital, Copenhagen, Denmark; 5 Centre of Excellence for Health, Immunity and Infections (CHIP), Rigshospitalet, Copenhagen, Denmark; 6 The National Board of Health, Nuuk, Greenland; 7 Ilulissat Regional Hospital, Ilulissat, Greenland; 8 Department of Clinical Medicine, University of Aarhus, Aarhus, Denmark; 9 Department of Internal Medicine, Queen Ingrid’s Hospital, Nuuk, Greenland

**Keywords:** COVID-19, Greenland, Exit screening, Travel measures, SARS-CoV-2 testing

## Abstract

**Background:**

During the early part of the COVID-19 pandemic, travel restrictions were implemented in Greenland to contain SARS-CoV-2 transmission. Despite their widespread use, empirical evidence supporting the effectiveness of travel restrictions is scarce. Greenland was in a unique position to maintain pre-departure testing among travellers, and we aimed to describe the effectiveness of pre-departure testing to inform the implementation of travel restrictions in future outbreaks.

**Methods:**

Our analysis included SARS-Cov-2 PCR test results from travellers, including children, arriving in Greenland from Denmark between June 15, 2020, and January 26, 2022. Additionally, we identified positive tests performed within 14 days post-arrival to Greenland in this population. We estimated the sensitivity of pre-departure screening by dividing the number of positive cases identified pre-departure by the sum of cases identified pre-departure and within 14 days post-arrival in Greenland.

**Results:**

Our analysis covered around 43% of all travellers who underwent pre-departure screening. Out of 26,406 SARS-CoV-2 PCR tests, the proportion of positive tests was 0.6%, which varied over time according to the incidence in Denmark. Overall sensitivity of pre-departure screening was 59% and decreased over time, reaching a minimum of 36% in January 2022. The overall proportion of positive PCR post-arrival tests among all travellers was 0.4%.

**Conclusions:**

Implementing pre-departure PCR testing for SARS-CoV-2 among travellers can be effective in the early stages of outbreaks, particularly in geographical regions like Greenland where community transmission had not yet occurred. Our findings suggest that pre-departure screening of travellers contributed to delaying community transmission in Greenland compared to most other countries, thereby reducing the COVID-19 disease burden.

**Supplementary Information:**

The online version contains supplementary material available at 10.1186/s12889-025-21844-y.

## Introduction

The COVID-19 pandemic led to numerous international and national restrictions to limit the importation or spread of the disease [1]. Consequently, there was a 70% decline in international tourist arrivals globally [2]. Similar travel measures have historically been employed during other outbreaks of influenza, severe acute respiratory syndrome (SARS), Middle East respiratory syndrome (MERS), and Ebola [3].

Despite widespread use in the early phase of the COVID-19 pandemic, the application of travel restrictions to contain outbreaks lacks evidence from existing literature [4]. Most studies are based on modelling assumptions built on premises that may differ from real-world situations, introducing uncertainties and potential inaccuracies in such model predictions. Thus, there is a need for studies based on empirical data to evaluate the effectiveness of travel measures [5].

It has been suggested that the combined use of public health and social measures (PHSMs), including travel measures, can limit the spread of infectious diseases, especially before community transmission begins [4, 6, 7]. A delayed time to community transmission is advantageous to prepare hospitals for increased patient intake and to increase the chance of available vaccine prophylaxis and treatment [8]. Travel measures are particularly effective in island states or isolated geographic regions like Greenland, where authorities can control all traffic in and out of the country [9, 10]. Nonetheless, the implementation of travel measures in island nations should be carefully considered, as they can affect the supply of essential items to the population, have serious economic consequences, and may be in conflict with civil rights [11].

Initially, the Greenlandic authorities recognised that the Arctic would be particularly vulnerable to COVID-19 due to the population’s non-immunity, the high prevalence of risk factors for severe disease such as obesity and chronic disease and crowded living conditions. Additionally, vast distances connected only by air or water transport made patient transportation challenging, and the healthcare system was at risk of becoming easily overburdened in case of many severely ill COVID-19 patients [12, 13]. In March 2020, at the beginning of the COVID-19 pandemic, the main transport route into Greenland was by air from Denmark. Additionally, there were weekly flights from Iceland to East Greenland and Nuuk. By sea, cargo ships travelled weekly from Denmark to Greenland with a limited number of passengers [13]. These unique geographical circumstances were advantageous, enabling the authorities to effectively control all incoming international travel. Consequently, the Greenlandic authorities quickly implemented strict travel measures combined with other temporary PHSMs, such as working-from-home policies and closure of most public spaces. Complete border closures were implemented on March 14, 2020, with exemptions for essential personnel such as healthcare staff. All travellers arriving in Greenland were also required to undergo a 14-day mandatory quarantine. Later, on June 15, mandatory pre-departure polymerase chain reaction (PCR) testing for travellers above 12 years of age, combined with a shorter 5-day mandatory quarantine and a PCR post-arrival test, was implemented [14]. It was not until October 2020 that children under the age of 12 were also required to provide a pre-departure PCR test.

Restrictions such as mandatory quarantine and post-arrival testing for SARS-CoV-2 in travellers as well as closure of international borders fluctuated throughout the early stages of the pandemic in response to the introduction of vaccines and the epidemiological situation in Greenland and Denmark [12]. However, pre-departure testing remained in place for most of the pandemic, as this measure was believed to be crucial in minimising the importation of the virus and thereby breaking potential transmission chains, by identifying asymptomatic travellers carrying SARS-CoV-2 [15]. By January 26, 2022, community transmission of SARS-CoV-2 had become a reality in Greenland, and at least 67% of the population was fully vaccinated against the virus [12], therefore the pre-departure testing mandate was lifted [16]. This led to the conclusion that pre-departure testing was no longer necessary at this stage of the pandemic. Additionally, the less virulent Omicron variant was predominant in Greenland at this time [12], resulting in a milder disease burden that could be managed within the health care system [13].

Greenland's pre-departure testing strategy required significant logistic and economic resources. The allocation of resources to testing facilities and personnel presumably acquired substantial costs, while the overall benefits for society and public health remain unexplored. A scientific study is needed to assess the effectiveness of such travel measures and provide guidance for future use not only in Greenland but also in other similar geographical regions. We hypothesise that the pre-departure testing (combined with other travel and PHSMs) significantly contributed to the delayed peak of SARS-CoV-2 transmission and the reduced burden of disease in Greenland [12, 13]. Therefore, we aimed to investigate the overall effectiveness of the pre-departure PCR testing for travellers to Greenland in limiting the transmission of SARS-CoV-2.

## Methods

We analysed the results of all pre-departure SARS-CoV-2 PCR tests in travellers from Denmark to Greenland, that were registered in the Corona Application Tool for Collaborating Hospitals (CATCH) treatment database [17] as well as post-arrival (within 14 days) PCR tests registered in Greenland from June 15, 2020, to January 26, 2022.

### Test facilities

The pre-departure screening involved a PCR test taken within 48 h before departure. A positive result prevented entry until the person tested negative or recovered from the infection, thus no longer being infectious. At the beginning of the pandemic, testing facilities were limited in Denmark, and all pre-departure testing of travellers to Greenland was conducted free of charge at one centre (Rigshospitalet University Hospital, Copenhagen, Denmark). The test results were registered in the CATCH treatment database which was administered by the Centre of Excellence for Health, Immunity and Infections (CHIP) at Rigshospitalet. Later, PCR testing became widely available to the public and travellers could receive free PCR tests in many other locations in Denmark. Initially, all PCR test results were reported to the Greenlandic airline company to ensure that all passengers tested negative before departure. Later in the pandemic, when COVID-19 immunity passports became available, travellers were able to provide proof of a negative test at the airport.

In Greenland, PCR testing for SARS-CoV-2 was not available until April 2020. Prior to that, samples had to be sent by air to Denmark, which had the necessary lab facilities. The Greenlandic health care system provided PCR tests free of charge. These tests were available to everyone in Greenland including travellers who were required to provide a negative post-arrival test throughout most of the pandemic.

### Data sources

Data from PCR test results of individuals travelling from Denmark to Greenland included information on unique personal identification number, name, intended date of travel to Greenland, and test date and result. For individuals with multiple tests registered on the same day, we allowed only one registration per person, and a positive result overruled any negative result. Furthermore, individuals with a comment linked to the test result specifying that the test was not conducted due to travelling to Greenland were also excluded.

Age, sex, and nationality of the travellers were identified based on the Danish/Greenlandic personal identification number.

Information on all positive post-arrival PCR test results obtained in Greenland was given by the Greenlandic National Board of Health. Data contained information on personal identification number, name, and test date. Information on the number of travellers to the Greenlandic international airports is publicly available at Statistics of Greenland [18].

### Statistical analysis

We used the Kruskal–Wallis test for continuous variables and Pearson’s Chi-squared test/Fisher’s exact test for categorical variables. For continuous non-parametric variables, we calculated a median with 25–75% interquartile range (IQR).

We calculated the total and monthly proportion of positive pre-departure PCR tests conducted on travellers by age from Denmark to Greenland. The number of conducted PCR tests for each period was compared with the total number of international passengers travelling to Greenland in the same period corresponding to the "proportion under surveillance". Secondly, we calculated the total and monthly proportion of positive PCR tests by age conducted in Greenland within 14 days from the pre-departure test to provide a measure of the extent to which the travel screening program did not capture SARS-CoV-2 infection before entry into Greenland, i.e., how many individuals likely had the infection upon entry but did not test positive by PCR test (false negatives). We assumed that a detected infection more than 14 days after arrival cannot be termed an imported infection but rather an infection obtained after entry into Greenland.

We calculated the sensitivity, including a 95% confidence interval (CI), of the pre-departure screening. For the purpose of this paper, we defined sensitivity as the total number of positive PCR tests identified by the pre-departure screening (true positives) relative to all identified positive PCR tests (the sum of pre-departure (true positives) and post-arrival PCR tests (false negatives)). We assumed that a positive post-arrival PCR test was caused by infection obtained before travel and not in Greenland. This ad hoc definition of sensitivity did not reflect the diagnostic sensitivity of the SARS-CoV-2 PCR test itself, but rather the ability of the pre-departure testing system to identify as many positive cases as possible among travellers. We used this measure to reflect the effectiveness of the pre-travel screening system.

Additionally, we calculated the negative predictive value (NPV) for the completeness of our results by dividing the total number of negative pre-departure PCR tests by the sum of the number of negative pre-departure and positive post-arrival PCR tests. We were unable to calculate the positive predictive value (PPV) due to lack of information on false positive cases.

Data analysis and visualisation were conducted using Microsoft Excel and R, version 4.2.3 [19].

## Results

The study included a total of 26,406 SARS-CoV-2 PCR tests conducted on travellers from Denmark to Greenland. The majority (99%) of travellers were of Danish or Greenlandic nationality. Age and sex distribution are shown in Table 1. The total number of registered pre-departure PCR tests corresponded to 43.1% (*n* = 26,406/61,243) of all tests conducted on international travellers to Greenland between June 2020 and January 2022 (Supplementary Table 1). In the period from July 2020 to May 2021, coverage of the pre-departure test was more than 50% (Supplementary Table 1).

**Table 1:** Descriptive statistics of travellers from Denmark to Greenland with a registered pre-departure SARS-CoV-2 PCR test from June 15, 2020, to January 26, 2022

	**Overall** **(*N* = 26406)**	**Travellers with a negative pre-departure SARS-CoV-2 PCR test** **(*N* = 26240)**	**Travellers with a positive pre-departure SARS-CoV-2 PCR test** **(*N* = 166)**	*p*-value*
**Age at pre-departure test in years**				0.006
Median (IQR)	45 (30–58)	45 (30–58)	37 (27–55)	
**Sex**				0.207
Female	12,341 (46.7%)	12,272 (46.8%)	69 (41.6%)	
Male	14,065 (53.3%)	13,968 (53.2%)	97 (58.4%)	
**Nationality**				0.799
Danish/Greenlandic	26,067 (98.7%)	25,904 (98.7%)	163 (98.2%)	
Non-Danish/Greenlandic	339 (1.3%)	336 (1.3%)	3 (1.8%)	

*IQR* Interquartile Range, *PCR* Polymerase chain reaction

^*^P-values were estimated using Kruskal’s Wallis test for continuous variables and Pearson’s Chi-squared test/ Fisher’s exact test for categorical variables

We found a median interval of 3 days (IQR 2–4 days) between date of pre-departure PCR test and intended date of travel to Greenland. Median interval between a negative pre-departure test and a positive post-arrival test was 8 days (IQR 6–12 days) with a distribution largely centred around 6–10 days (Supplementary Fig. 1).

**Fig. 1:**
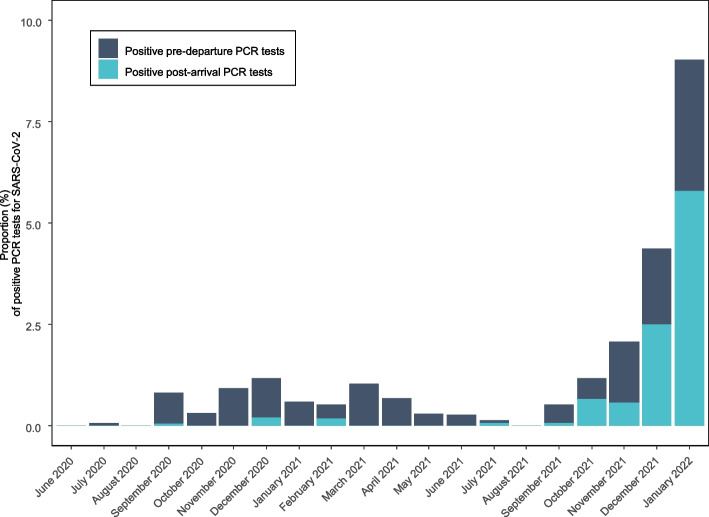
Proportion (%) of positive SARS-CoV-2 pre-departure PCR tests (dark blue) and post-arrival PCR tests (light blue) of travellers to Greenland from Denmark in the period June 15, 2020, to January 26, 2022

Of all tested individuals, 166 tested positive for SARS-CoV-2 (0.6%) pre-departure to Greenland (Fig. 1). The proportion of positive SARS-CoV-2 pre-departure tests did not differ significantly between travellers aged ≤ 12 years and those aged > 12 years (*p*-value = 0.529) (Supplementary Table 1). The proportion of positive pre-departure tests increased over time since the implementation of the testing program, reaching a maximum of 3.6% in January 2022 (Fig. 1 and Supplementary Table 1). The overall proportion of positive post-arrival PCR tests among travellers to Greenland was 0.4%, with no significant difference between travellers aged ≤ 12 years and those aged > 12 years (*p*-value = 0.935) (Supplementary Table 2). We found a similar pattern with an increasing proportion of positive post-arrival tests over time (Supplementary Table 2).

**Fig. 2:**
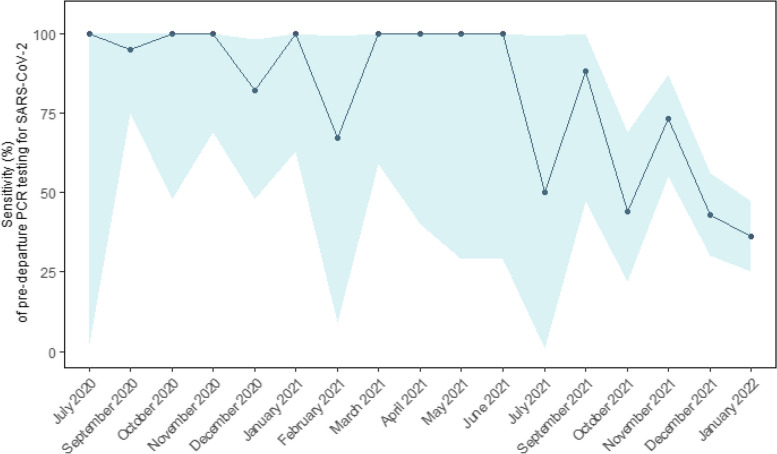
Sensitivity (%) of SARS-CoV-2 pre-departure PCR testing of travellers to Greenland from Denmark in the period June 15, 2020, to January 26, 2022. The points show the sensitivity estimate for each month with a shaded area corresponding to a 95% confidence interval. Months with missing data are not shown

Overall sensitivity of the pre-departure PCR testing was 59.7%. Sensitivity fluctuated throughout the pandemic, ranging between 35.8% (in January 2022 when pre-departure screening was discontinued) and 100% (Fig. 2 and Supplementary Table 3). Similarly, the NPV ranged between 93.8% (in January 2022) and 100% during the study period (Supplementary Table 3).

**Fig. 3:**
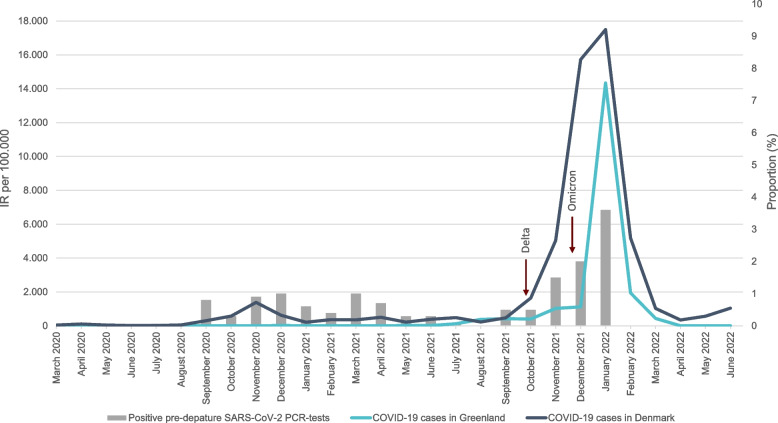
Illustration of the proportion of positive pre-departure SARS-CoV-2 PCR tests of travellers to Greenland from Denmark by month/year to the incidence rates (IR) per 100.000 inhabitants in Greenland (light blue line) and Denmark (dark blue line) from March 2020 to June 2022. Arrows indicate when the Delta and Omicron variants were predominant in Greenland

**Fig. 4:**
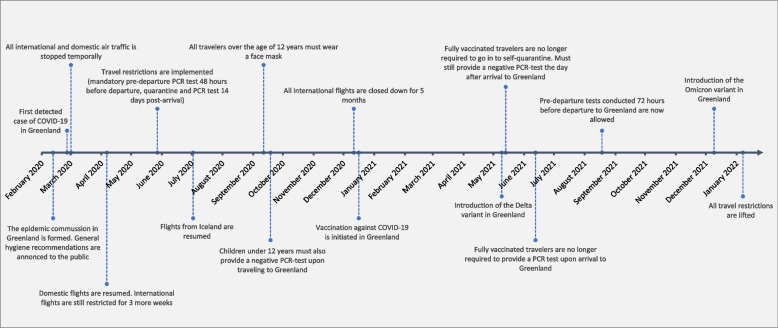
Timeline of selected COVID-19 restrictions implemented in Greenland from March 2020 to January 2022. Source: Greenlandic news media Sermitsiaq and KNR

Figure 3 illustrates how the proportion of positive pre-departure tests follows the surge in case numbers (IR per 100,000) in Denmark in particular with a sharp increase in the early autumn of 2021. In addition, this aligns with the changes in COVID-19 restrictions in Greenland as shown in Fig. 4, where several travel restrictions were dropped during the summer/fall of 2021.

## Discussion

### Main findings

We found that pre-departure testing was equally effective in detecting positive SARS-CoV-2 cases both in travellers aged ≤ 12 years and those aged > 12 years, highlighting the importance of testing both adults and children. Additionally, our results indicate that a pre-departure test taken up to 72 h prior to departure is sufficient for detecting travellers infected with SARS-CoV-2. We found a high sensitivity and NPV of pre-departure PCR testing of SARS-CoV-2 in travellers to Greenland at the beginning of the pandemic. However, as the pandemic progressed, and especially when community transmission occurred in Greenland, both the sensitivity and NPV of pre-departure screening decreased substantially. This suggests that such travel measures might be most effective early in pandemics or outbreaks before case numbers are high in the country of arrival. Our results show that a considerable portion of the travellers who had been classified as false negatives tested positive for SARS-CoV-2 between 12–14 days after arriving in Greenland. This could have influenced the estimated sensitivity and NPV, as these travellers might have contracted the virus during the flight or after their arrival (with a short incubation period), rather than before departure. As a result, they may be incorrectly identified as false negatives, leading to an underestimation of the sensitivity and NPV.

The decreasing effectiveness correlated with the removal of other travel measures in the summer/fall of 2021, such as the mandatory quarantine, testing of travellers post-arrival and the relaxed requirement for a pre-departure test within 72 h before departure. This suggests that pre-departure testing is more effective when implemented in combination with other travel measures and PHSMs rather than as a solitary measure. The findings of our study emphasize the value of dual-testing approach, combining pre-departure and post-arrival tests, to prevent the introduction of SARS-CoV-2 into an immune-naïve population like Greenland. Similar findings have been reported in other studies, which also underscore the importance of timely implementation and combining travel measures with PHSMs to effectively control outbreaks [6, 20]. Furthermore, studies generally recommend a test-based screening of travellers rather than a symptom/exposed-based screening which has been shown to have very limited effectiveness [1, 4, 20–22]. The World Health Organization (WHO) also recommended test-based screening of travellers during the COVID-19 pandemic, despite its high cost and resource demands [11].

### Secondary impacts of travel measures

A secondary benefit of a pre-departure testing strategy is that it may discourage symptomatic individuals from travelling, enhance the confidence of healthy travellers, and provide an opportunity to inform and educate travellers about the disease [22]. This may also explain the very low proportion of positive pre-departure tests we found in this study, as sick individuals might have postponed their travels to Greenland. Unfortunately, we did not have information on the number of symptomatic travellers or how many people who were supposed to travel cancelled their trips due to symptoms and therefore did not get tested.

As our results illustrate, the effectiveness of pre-departure testing, and presumably other travel measures such as post-arrival testing, decreased over time when the epidemiological situation changed in both Denmark and Greenland, with a sharp increase in case numbers in the autumn of 2021 and the introduction of SARS-CoV-2 variants (Delta and Omicron) with a higher transmission rate [12]. As mentioned, the Greenlandic authorities decided to discontinue the requirement for pre-departure testing, as the effectiveness of this screening strategy appeared negligible at that time [16]. Other studies evaluating the effect of travel measures during the COVID-19 pandemic also highlighted the importance of adapting the use of travel measures to the countries' individual epidemiological trends [6]. Our results could act as a surrogate for SARS-CoV-2 case numbers in the country of departure, in this case Denmark, thereby aiding the mentioned adaptation. This is particularly important when considering the possible negative impact of travel measures on areas such as loss of income due to decreased tourism, major economic and resource consequences, and the individual's possibilities to return to their home country [23, 24]. For pre-departure testing specifically, the huge demand for human, laboratory, logistical and economic resources may reduce the amount of resources available for the preparedness of other PHSMs that are needed during an outbreak [22].

### Strengths and limitations

Our study contributes to assessing one of several protective measures implemented during the COVID-19 pandemic. Notably, our analysis was limited to approximately 43% of the pre-departure PCR tests administered to travellers bound for Greenland during the study period. This incomplete coverage introduces some uncertainty into our strategy's effectiveness and the broader applicability of our results. The somewhat limited surveillance proportion of 43% may be caused by several factors. For instance, data on international travellers by air to Greenland included both adults and children, although children were only required to provide a negative pre-departure test from around October 2020. Additionally, introduction of the COVID-19 immunity passport in May 2021 in Denmark allowed travellers to use it freely, resulting in some test results not being registered in the CATCH treatment database. However, incomplete data is often a limitation when using retrospectively collected registry data, as in this study, and this factor should be considered when interpreting the results.

Furthermore, a significant limitation of this study is that it was not possible to adequately account for the impact of other PHSMs or travel-related measures implemented simultaneously with pre-departure testing. Finally, this study does not represent a full evaluation of the screening programme, but it provides important data on the performance, which is essential for planning similar endeavours. It is, for example, misleading to estimate the number needed to screen to identify one case, since a simple calculation (1/0.006) will underestimate the true effectiveness, since pre-departure testing may discourage travellers with symptoms or with known exposure from being tested. Hence the real effectiveness may be greater than the numbers suggest.

Nevertheless, our findings indicate that the pre-departure testing strategy, combined with other travel measures, significantly delayed the entry of COVID-19 into Greenland relative to many other countries. This delay provided authorities with additional time to implement other interventions such as vaccines, which we believe ultimately contributed to a reduced burden of disease in Greenland. Our study provides valuable knowledge of outbreak preparedness for Greenland and similar populations and geographical areas, including island communities and regions with limited entry routes. This knowledge can be instrumental in developing national strategies to mitigate infection, whether facing a new wave of COVID-19 or another emerging or known virus with a similar transmission pattern.

## Conclusions

In summary, we found that pre-departure PCR testing for SARS-CoV-2 of travellers of all ages to Greenland was effective in identifying infected travellers during the early stages of the COVID-19 pandemic. However, as community transmission increased in Greenland, the effectiveness of pre-departure screening decreased. This suggests that pre-departure testing is most effective when implemented early in outbreaks. Additionally, combining pre-departure testing with other travel measures such as post-arrival testing and PHSMs enhances its effectiveness. While our study provides valuable information, limitations such as incomplete evaluation and the impact of concurrent COVID-19 PHSMs should be considered. Overall, pre-departure testing played a noteworthy role in delaying COVID-19 entry into Greenland and can be applied in case of future outbreaks in Greenland and similar geographical areas and populations.

## Supplementary Information

Supplementary Material 1.

## Data Availability

The datasets generated and/or analysed during the current study are not publicly available due to Danish/Greenlandic data protection law and ethical considerations. Data are however available from the authors upon reasonable request and with permission of the Greenland Research Ethics Committee as well as the Centre of Excellence for Immunity, Health and Infections (CHIP), Rigshospitalet.

## Abbreviations

SARS: Severe acute respiratory syndrome
MERS: Middle East respiratory syndrome
PHSM: Public health and social measures
PCR: Polymerase chain reaction
SARS-CoV-2: Severe acute respiratory syndrome coronavirus 2
CATCH: Corona Application Tool for Collaborating Hospitals
CHIP: Centre of Excellence for Health, Immunity and Infections
IQR: Interquartile range
CI: Confidence interval
NPV: Negative predictive value
PPV: Positive predictive value
IR: Incidence rate
WHO: World Health Organization
