# Supplementary material for: Pre-departure PCR testing of travellers for SARS-CoV-2 was an effective tool in limiting transmission in Greenland during the early phases of the COVID-19 pandemic
Source: BMC Public Health. 2025 Feb 12;25:585. doi: 10.1186/s12889-025-21844-y (PMC11823211; doi:10.1186/s12889-025-21844-y)
Supplement: Supplementary file 1 — Supplementary Material 1. [file 12889_2025_21844_MOESM1_ESM.docx]

**Supplementary material**

**Supplementary Table 1**

Proportion of positive pre-departure SARS-CoV-2 PCR tests in travellers to Greenland from Denmark from June 15, 2020, to January 26, 2022, grouped by age ≤ 12 years.

| **Month/year of test** | **Overall** | **Proportion of positive pre-departure SARS-CoV-2 PCR tests (%) in travellers ≤ 12 years** | **Proportion of positive pre-departure SARS-CoV-2 PCR tests (%) in travellers > 12 years** | **Proportion under surveillance^c^ (%)** |
| --- | --- | --- | --- | --- |
| Total N | 166/26406 (0.6) | 14/1822 (0.8) | 152/24584 (0.6) | 26406/61243* (43.1) |
| **2020** | | | |  |
| June^a^ | 0/501 (0) | 0/40 (0) | 0/461 (0) | 501/1690* (29.6) |
| July | 1/1704 (0.06) | 0/156 (0) | 1/1548 (0.06) | 1704/2984 (57.1) |
| August | 0/2776 (0) | 0/166 (0) | 0/2610 (0) | 2776/4054 (68.5) |
| September | 19/2461 (0.8) | 0/114 (0) | 19/2347 (0.8) | 2461/3747 (65.7) |
| October | 5/1622 (0.3) | 1/104 (1.0) | 4/1518 (0.3) | 1622/2729 (59.4) |
| November | 10/1072 (0.9) | 1/63 (1.6) | 9/1009 (0.9) | 1072/1389 (77.2) |
| December | 9/928 (1.0) | 0/57 (0) | 9/871 (1.0) | 928/2492 (37.2) |
| **2021** | | | |  |
| January | 8/1345 (0.6) | 0/110 (0) | 8/1235 (0.6) | 1345/1215 (110.7) |
| February | 2/563 (0.4) | 0/19 (0) | 2/544 (0.4) | 563/770 (73.1) |
| March | 7/674 (1.0) | 0/26 (0) | 7/648 (1.1) | 674/1025 (65.8) |
| April | 4/583 (0.7) | 0/30 (0) | 4/553 (0.7) | 583/1268 (46.0) |
| May | 3/998 (0.3) | 0/55 (0) | 3/943 (0.3) | 998/1819 (54.9) |
| June | 3/1114 (0.3) | 0/72 (0) | 3/1042 (0.3) | 1114/3125 (35.6) |
| July | 1/1508 (0.07) | 0/171 (0) | 1/1337 (0.07) | 1508/4735 (31.8) |
| August | 0/1806 (0) | 0/175 (0) | 0/1631 (0) | 1806/5365 (33.7) |
| September | 7/1537 (0.5) | 1/91 (1.1) | 6/1446 (0.4) | 1537/5554 (27.7) |
| October | 8/1512 (0.5) | 2/109 (1.8) | 6/1403 (0.4) | 1512/5375 (28.1) |
| November | 24/1553 (1.5) | 4/99 (4.0) | 20/1454 (1.4) | 1553/4440 (35.0) |
| December | 26/1333 (2.0) | 2/96 (2.1) | 24/1237 (1.9) | 1333/4602 (29.0) |
| **2022** | | | |  |
| January^b^ | 29/816 (3.6) | 3/69 (4.4) | 26/747 (3.5) | 816/2865* (28.5) |

^a^as of June 15, 2020

^b^until January 26, 2022

^c^the number of pre-departure SARS-CoV-2 PCR tests conducted each month compared to the number of passengers travelling by aircraft to Greenland during the same period.

*includes the number of airline passengers for the entire month of June 2020 and January 2022.

**Supplementary Figure 1**

Distribution of time (days) between a SARS-CoV-2 negative pre-departure PCR test and positive post-arrival PCR test among travellers to Greenland from Denmark from June 15, 2020, to January 26, 2022.

**
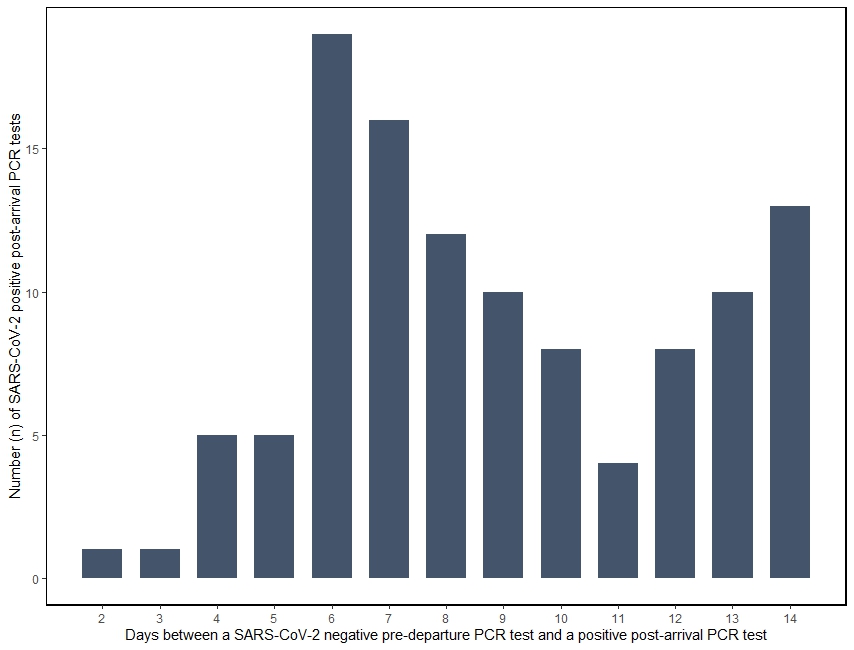
**

**Supplementary Table 2**

Proportion of positive post-arrival SARS-CoV-2 PCR tests in travellers to Greenland from Denmark from June 15, 2020, to January 26, 2022, grouped by age ≤ 12 years.

| **Month/year of test** | **Overall** | **Proportion of positive post-arrival^c^ SARS-CoV-2 PCR tests (%) in travellers ≤ 12 years** | **Proportion of positive post-arrival^c^ SARS-CoV-2 PCR tests (%) in travellers > 12 years** |
| --- | --- | --- | --- |
| Total N | 112/26240 (0.4) | 7/1808 (0.4) | 105/24432 (0.4) |
| **2020** | | | |
| June^a^ | 0/501 (0) | 0/40 (0) | 0/461 (0) |
| July | 0/1703 (0) | 0/156 (0) | 0/1547 (0) |
| August | 0/2776 (0) | 0/166 (0) | 0/2610 (0) |
| September | 1/2442 (0.04) | 0/114 (0) | 1/2328 (0.04) |
| October | 0/1617 (0) | 0/103 (0) | 0/1514 (0) |
| November | 0/1062 (0) | 0/62 (0) | 0/1000 (0) |
| December | 2/919 (0.2) | 0/57 (0) | 2/862 (0.2) |
| **2021** | | | |
| January | 0/1337 (0) | 0/110 (0) | 0/1227 (0) |
| February | 1/561 (0.2) | 0/19 (0) | 1/542 (0.2) |
| March | 0/667 (0) | 0/26 (0) | 0/641 (0) |
| April | 0/579 (0) | 0/30 (0) | 0/549 (0) |
| May | 0/995 (0) | 0/55 (0) | 0/940 (0) |
| June | 0/1111 (0) | 0/72 (0) | 0/1039 (0) |
| July | 1/1507 (0.07) | 0/171 (0) | 1/1336 (0.07) |
| August | 0/1806 (0) | 0/175 (0) | 0/1631 (0) |
| September | 1/1530 (0.07) | 1/90 (1.1) | 0/1440 (0) |
| October | 10/1504 (0.7) | 0/107 (0) | 10/1397 (0.7) |
| November | 9/1529 (0.6) | 2/95 (2.1) | 7/1434 (0.5) |
| December | 35/1307 (2.7) | 0/94 (0) | 35/1213 (2.9) |
| **2022** | | | |
| January^b^ | 52/787 (6.6) | 4/66 (6.1) | 48/721 (6.7) |

^a^as of June 15, 2020

^b^until January 26, 2022

^c^includes SARS-CoV-2 PCR-tests conducted up to 14 days post-arrival to Greenland

**Supplementary Table 3**

Sensitivity and negative predictive value (NPV) of pre-departure SARS-CoV-2 PCR testing of travellers to Greenland from Denmark from June 15, 2020, to January 26, 2022.

| **Month/year of test** | **Sensitivity %**  **(95% CI)** | **NPV %**  **(95% CI)** |
| --- | --- | --- |
| Total N | 59.7 (53.7-65.5) | 99.6 (99.5-99.7) |
| **2020** | |  |
| June^a^ | - | - |
| July | 100 (2.5-100) | 100 (100-100) |
| August | - | - |
| September | 95 (75.1-99.9) | 99.9 (99.9-100) |
| October | 100 (47.8-100) | 100 (100-100) |
| November | 100 (69.2-100) | 100 (100-100) |
| December | 81.8 (48.2-97.7) | 99.8 (99.5-100) |
| **2021** | |  |
| January | 100 (63.1-100) | 100 (100-100) |
| February | 66.7 (9.4-99.2) | 99.8 (99.5-100) |
| March | 100 (59.0-100) | 100 (100-100) |
| April | 100 (39.8-100) | 100 (100-100) |
| May | 100 (29.2-100) | 100 (100-100) |
| June | 100 (29.2-100) | 100 (100-100) |
| July | 50 (1.3-98.7) | 99.9 (99.8-100) |
| August | - | - |
| September | 87.5 (47.3-99.7) | 99.9 (99.8-100) |
| October | 44.4 (21.5-69.2) | 99.3 (98.9-99.7) |
| November | 72.7 (54.5-86.7) | 99.4 (99.0-99.8) |
| December | 42.6 (30.0-55.9) | 97.4 (96.5-98.2) |
| **2022** | |  |
| January^b^ | 35.8 (25.4-47.2) | 93.8 (92.2-95.4) |

^a^as of June 15, 2020

^b^until January 26, 2022

CI: confidence interval
